# Knockout of the *hsd11b2* Gene Extends the Cortisol Stress Response in Both Zebrafish Larvae and Adults

**DOI:** 10.3390/ijms222212525

**Published:** 2021-11-20

**Authors:** Antonia Theodoridi, Alberto Dinarello, Lorenzo Badenetti, Michail Pavlidis, Luisa Dalla Valle, Aleka Tsalafouta

**Affiliations:** 1 Department of Biology, University of Crete, P.O. Box 2208, 714 09 Heraklion, Greece; Tonia.theod@gmail.com (A.T.); pavlidis@uoc.gr (M.P.); 2 Department of Biology, University of Padova, 35121 Padova, Italy; alberto.dinarello@phd.unipd.it (A.D.); lorenzo.badenetti@phd.unipd.it (L.B.)

**Keywords:** zebrafish, stress, glucocorticoid, cortisol, *hsd11b2*, CRISPR/Cas9, LMR-L/D, VSRA

## Abstract

The Hsd11b2 enzyme converts cortisol into its inactive form, cortisone and regulates cortisol levels, in particular in response to stress. Taking advantage of CRISPR/Cas9 technology, we generated a *hsd11b2* zebrafish mutant line to evaluate the involvement of this gene in stress response regulation. The absence of a functional Hsd11b2 affects survival of zebrafish, although homozygous *hsd11b2^−/−^* mutants can reach adulthood. Reproductive capability of *hsd11b2^−/−^* homozygous adult males is almost completely abrogated, while that of females is reduced. Interestingly, basal cortisol levels and glucocorticoid-dependent transcriptional activities are not affected by the mutation. In agreement with basal cortisol results, we also demonstrated that basal response to light (LMR-L/D) or mechanical (VSRA) stimuli is not significantly different in wild-type (*hsd11b2^+/+^*) compared to mutant larvae. However, after exposure to an acute stressor, the cortisol temporal patterns of synthesis and release are prolonged in both 5 days post fertilization larvae and one-year-old adult *hsd11b2^−/−^* zebrafish compared to wild-type siblings, showing at the same time, at 5 dpf, a higher magnitude in the stress response at 10 min post stress. All in all, this new zebrafish model represents a good tool for studying response to different stressors and to identify mechanisms that are induced by cortisol during stress response.

## 1. Introduction

Vertebrates respond to stress via activation of the Hypothalamic–Pituitary–Adrenal (HPA) axis, leading to the primary, secondary and tertiary stress responses [1,2]. In teleost fish, like zebrafish (*Danio rerio*), the HPA corresponds to the Hypothalamic–Pituitary–Interrenal (HPI) axis. As in the case of other animals, the HPI activation leads to the release of catecholamines and cortisol as the primary response, adjustments in physiology and metabolism as the secondary response and, finally, changes at the whole animal or population level as part of the tertiary response [3,4]. Cortisol, both in humans and teleosts, is the primary glucocorticoid involved in stress response. Cortisol concentration increases in the bloodstream during stress, with magnitude and time patterns specific for each species [5]. Adult zebrafish show a dynamic response to stress with a very rapid and prolonged increase in whole-body cortisol concentrations, starting at around 15 min and returning to basal levels at about 2 h following exposure to acute stressors [6].

Elevation of cortisol after stressor exposure during early life can alter the physiology and behavior of animals [7,8]. In zebrafish, HPI axis activation occurs only after hatch, and the fish can respond to the stress event in terms of cortisol alterations starting from 72 h post fertilization (hpf) [9,10]. The cortisol stress response during early development has also been studied in zebrafish in which exposure of 5-days-post-fertilization (dpf) larvae pools to acute swirling stress elicits cortisol release peaking 5 min after stress and returning to resting levels 30 min after stress exposure [11]. In the studies investigating the larval stress cortisol levels, it was found that the duration of the stressor highly affects the response observed [11,12,13]. However, all these studies measure the cortisol levels of pooled larval samples and not individual whole-body cortisol concentrations.

The biosynthesis of cortisol in teleosts follows almost the same steroidogenic pathway as in higher vertebrates, including humans, with key players being the enzyme 11β-Hydroxylase (P45011c1), which participates in corticoid synthesis and the 11β-Hydroxysteroid dehydrogenase type 2 (Hsd11b2), which regulates cortisol bioavailability by converting this steroid into its inactive form and preventing, in human, non-correct activation of the mineralocorticoid receptor [14]. Studies in *Dicentrarchus labrax* have revealed a significant correlation between whole-body cortisol and mRNA transcript levels for *cyp11c1* and *hsd11b2* [15], confirming that the Hsd11b2 enzyme has a significant role in regulating the cortisol levels [16]. Moreover, in fish, Hsd11b2 is also required for the production of the 11-ketotestosterone (11KT), that is the major active androgen in male fish reproduction [17]. In contrast with mammals, zebrafish do no possess Hsd11b1, so it is not able to perform the keto-reduction that convert cortisone in cortisol [17]. Lacking the possibility to locally increase cortisol levels, de novo synthesis of cortisol is essential in zebrafish for GC signaling and 11β-Hsd2 plays a fundamental role in regulating cortisol access to Glucocorticoid Receptor and Mineralocorticoid Receptor [17].

In humans, mutations in the *HSD11B2* gene are responsible for a rare autosomal recessive disorder called apparent mineralocorticoid excess (AME) syndrome [18]. In AME patients, MR is overstimulated by an excess of cortisol leading to sodium retention with consequent hypokalemia, hypernatremia, and hypertension without elevated levels of aldosterone and renin [19]. In mice, knockout of *Hsd11b2* gene leads to high mortality soon after birth, and surviving mice show hypertension, hypokalemia and hypotonic polyuria [20,21].

In placenta, *HSD11B2* enzyme protects the fetus from active GCs derived from the mother. In the absence of *HSD11B2* activity the excessive presence of cortisol can induce intrauterine growth restriction (IUGR), increased cardiovascular risk as well as neuroendocrinology and anxiety disorders later in life [22]. In zebrafish, pre-vitellogenic follicles upregulate *hsd11b2* after exposure to cortisol excess, suggesting the presence of a similar mechanism to protect embryo from abnormal levels of cortisol [23]. Since the advent of CRISPR/Cas9-targeted mutagenesis a great number of zebrafish mutant lines have been generated to improve our knowledge of glucocorticoid biology and functions, as reviewed in Dinarello and collaborators [24]. However, despite this growing number of studies, little is known about how the deactivation of Hsd11b2 affects zebrafish in terms of larval development, endocrine regulation of the stress response and behavior. Therefore, this study aims to investigate the physiological and behavioral effects of inactivation of Hsd11b2 enzyme in larvae. To this end we generated and characterized a mutant knockout zebrafish line for this gene (*hsd11b2^ia33/ia33^* hereafter *hsd11b2^−/−^*). WT (*hsd11b2^+/+^*) and mutant (*hsd11b2^−/−^*) larvae were exposed to an acute stress and the cortisol temporal patterns were determined in individual larvae [25]. Moreover, the cortisol stress response was also evaluated in mutant and WT adult zebrafish. Additionally, we analyzed the locomotor response of both WT and mutant larvae to alternating Light/Dark periods (LMR-L/D) [26,27,28] and the escape response and its habituation by applying the Vibrational Startle Response Assay (VSRA) [29,30].

## 2. Results

### 2.1. Characterization of hsd11b2 Mutant Zebrafish Line

**Figure 1:**
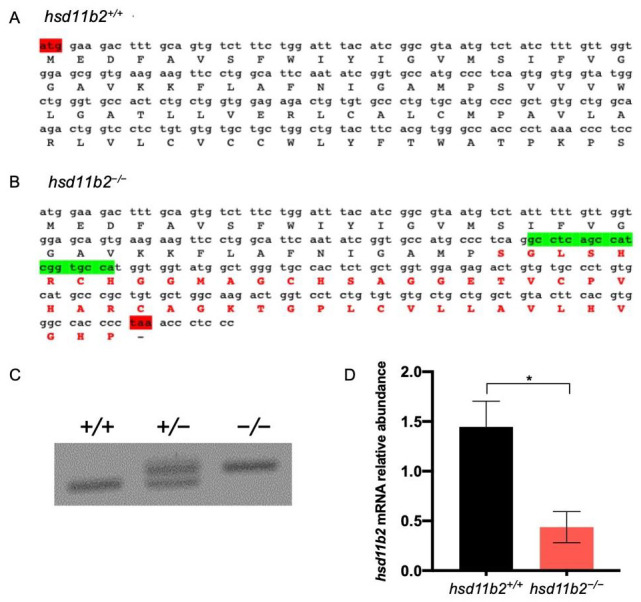
Generation of *hsd11b2* mutant zebrafish line. (**A**) Partial (from the start codon) nucleotide and aminoacidic sequence of *hsd11b2* in WT animals. (**B**) Partial (from the start codon) nucleotide and aminoacidic sequence of *hsd11b2* in mutant animals showing in green the 19-nt insertion. (**C**) Representative picture of *hsd11b2^+/+^,* *hsd11b2^+/−^* and *hsd11b2^−/−^* genotyping in 2% agarose gel. (**D**) RT-qPCR of *hsd11b2* transcripts in *hsd11b2^+/+^* and *hsd11b2^−/−^* 5-dpf larvae. *n* = 4 independent biological replicas. Mean ± SEM. Statistical significance was determined by Student’s *t*-test. * *p* < 0.05.

The *hsd11b2* mutant zebrafish line generated with CRISPR/Cas9 technology is characterized by an insertion of 19 nucleotides in the first coding exon of the *hsd11b2* gene (Figure 1). Homozygous and heterozygous mutants can be easily recognized by electrophoresis on 2% agarose gel of the PCR products obtained with the genotyping primers. The insertion determines a frameshift mutation that leads to the incorrect termination of translation before the functional domains of the enzyme. The non-functional, truncated protein supposedly encoded by the mutant contains 83 amino acids, of which the first 35 aa belong to the wild-type (WT) sequence of zebrafish Hsd11b2 enzyme, while the other 48 aa are determined by the loss of the correct coding frame.

**Figure 2:**
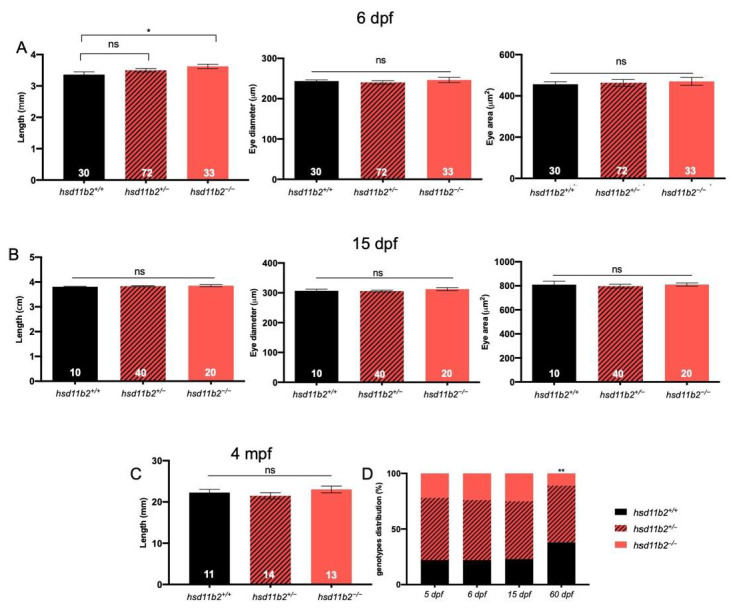
Morphometric analysis of *hsd11b2* mutant zebrafish line. (**A**) Measurements of body length, eye diameter and eye area of 6-dpf *hsd11b2^+/+^,* *hsd11b2^+/−^* and *hsd11b2^−/−^* larvae. (**B**) Measurements of body length, eye diameter and eye area of 15-dpf *hsd11b2*^+/+^, *hsd11b2*^+/−^ and *hsd11b2*^−/−^ larvae. (**C**) Measurements of body length of 3-mpf *hsd11b2^+/+^,* *hsd11b2^+/−^* and *hsd11b2^−/−^* fish. (**D**) Survival of *hsd11b2^+/+^,* *hsd11b2^+/−^* and *hsd11b2^−/−^* animals at 5, 6, 15 and 60 dpf. Mean ± SEM. significant. Student’s *t*-test was used to evaluate the morphometric differences and X^2^ test was used to evaluate the differences of genotype distribution compared to the expected ratios. * *p* < 0.05; ** *p* < 0.01; ns = not significant.

The significant downregulation of *hsd11b2* expression in *hsd11b2^−/−^* compared to *hsd11b2^+/+^* siblings should rely on non-sense mediated mRNA decay (NMD) mechanisms that are usually triggered in mutant lines [31]. The genetic abrogation of the Hsd11b2 activity determines a slight but significant increase of body length at 6 dpf. However, this effect cannot be detected at 15 dpf or at 3 months post fertilization (mpf) (Figure 2). We did not see any differences in eye diameter nor eye area between *hsd11b2^+/+^* and *hsd11b2^−/−^* siblings. Mutants do not show any abrupt phenotypes and they are completely indistinguishable from WT siblings from a morphological point of view both at larval and adult stage. On the contrary, the absence of a functional Hsd11b2 enzyme affects the survival of larvae since we observed a significant reduction in the % survival of homozygous mutants (11%) compared to the expected one (25%) at 60 dpf. At the same time, we could not see significant differences between observed and expected percentages of *hsd11b2^+/+^* and *hsd11b2^+/^*^−^ animals (Figure 2).

**Figure 3:**
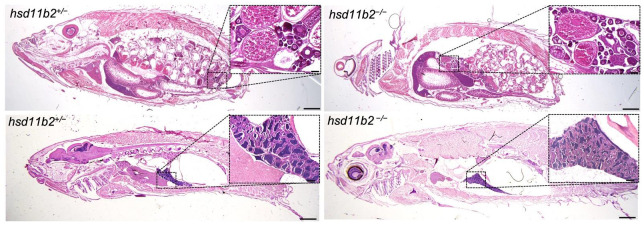
Histological analysis of 8-mpf female (**top panels**) and males (**bottom panels**) hsd11b2^+/−^ and hsd11b2^−/−^ zebrafish (scale bar = 1 mm). Magnification of ovaries (**top panels**) and testis (**bottom panels**) (scale bar = 100 um).

Crosses between heterozygotes were performed to generate *hsd11b2^+/+^* and *hsd11b2^−/−^* sibling larvae and adults. Indeed, the silencing of this gene appears to influence the reproductive capacity of mutant males which were unable to reproduce even with WT or *hsd11b2^+/−^* females. Conversely, reproductive capabilities of mutant females were reduced but not abolished when crossed with WT or *hsd11b2^+/−^* males. A preliminary histological analysis was performed on 8-mpf heterozygous and mutant individuals. Since the problem was related to reproduction, we focused our attention to the gonads, but we did not find evident alterations in either female’s or male’s gonads of 8-mpf mutants (Figure 3). At least at this stage, the testis of both genotypes appeared well organized with evident seminiferous tubules, containing cysts of all spermatogenic stages and mature sperm. Ovaries of both genotypes contained predominately vitellogenic and mature oocytes (Figure 3).

### 2.2. Single-Larva Whole-Body Cortisol and Stress Response

**Figure 4:**
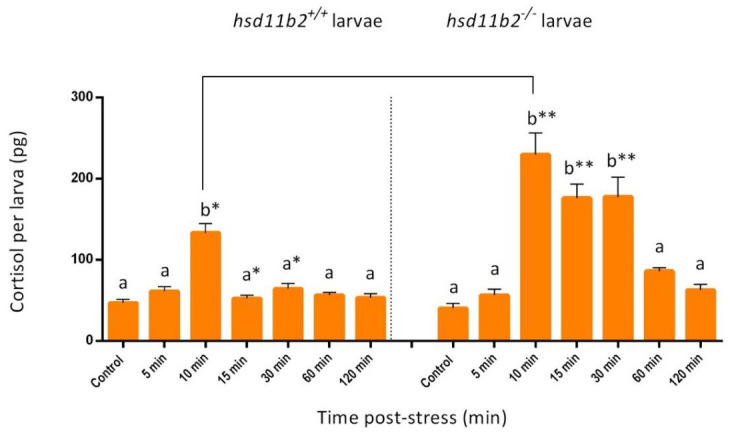
Effect of acute stress application on whole-body cortisol concentrations of individual larvae at 5 dpf, as depicted by bar graphs. Measurements of 10 individual *hsd11b2^+/+^* (WT) and 10 individual *hsd11b2^−/−^* (mutant larvae) was performed prior to (control) and 5, 10, 15, 30, 60 and 120 min after the stress application. Values are means ± SEM. Means with different letters differ significantly from one another (*p* < 0.001). Asterisks show statistically significant differences between the two groups (*p* < 0.001). Holm-Sidak comparison test was applied to identify groups that were significantly different. * *p* < 0.05, ** *p* < 0.01. Data for the two different genotypes are separated by the dot line in the graph.

The cortisol stress response after application of an acute stress was evaluated at 5 dpf in both *hsd11b2^+/+^* and *hsd11b2^−/−^* mutant zebrafish as depicted in Figure 4. To this end, larvae were subjected to an acute stress, and whole-body cortisol was measured prior to (0 min) and 5 min, 10 min, 15 min, 30 min, 60 min and 120 min after application of the stressor. For the *hsd11b2^+/+^* group, a statistically significant (*p* < 0.001) increase in whole-body cortisol concentrations was observed at 10 min post stress. More specifically, cortisol concentration was higher after 5 min (61.6 pg/larva ± 19.5 SEM) with respect to basal level at time 0 (47.1 pg/larva ± 14.9 SEM) and showed a peak at 10 min post stress (133.4 pg/larva ± 42.2 SEM). Cortisol returned to resting levels after 15 min post stress (52.7 pg/larva ± 16.7 SEM) and remained stable until 120 min post stress (53.3 pg/larva ± 16.9 SEM) (Figure 4).

On the other hand, a statistically significant (*p* < 0.001) higher peak at 10 min post-stress (230.0 pg/larva ± 72.8 SEM) and a prolonged duration (up to 30 min) of high whole-body cortisol concentrations, were observed in the *hsd11b2^−/−^* mutant compared to the *hsd11b2^+/+^* zebrafish. In this case, although whole-body cortisol basal values at time 0 (40.6 pg/larva ± 12.8 SEM) increased to reach peak levels at 10 min post stress (230.0 pg/larva ± 72.8 SEM) as in the case of the WT genotype, the stress response in terms of cortisol concentrations remained at high levels even at 15 min (176.3 pg/larva ± 55.8 SEM) and 30 min (177.9 pg/larva ± 56.3 SEM), to gradually fall at 60 min post stress (86.4 pg/larva ± 27.3 SEM) and to reach resting values only at 120 min post stress (63.1 pg/larva ± 20.0 SEM) (Figure 4).

**Figure 5:**
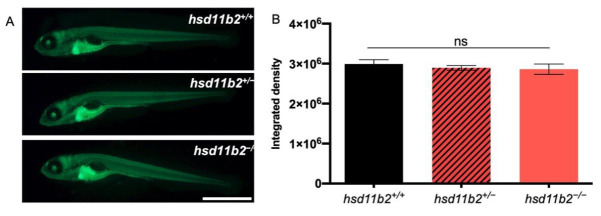
Analysis of GC-dependent transcriptional activities. (**A**) Representative pictures of 5-dpf *hsd11b2^+/+^*, *hsd11b2^+/−^,* and *hsd11b2^−/−^* larvae in *Tg(9xGCRE-HSV.Ul23:EGFP)^ia20^* transgenic background. (**B**) Fluorescence quantification of 5-dpf *hsd11b2^+/+^*, *hsd11b2^+/−^,* and *hsd11b2^−/−^* larvae in *Tg(9xGCRE-HSV.Ul23:EGFP)^ia20^* transgenic background. Scale bar = 1 mm. Statistical significance was determined by Student’s *t*-test. ns = not significant. Mean ± SEM.

The absence of significant differences in basal cortisol concentration between *hsd11b2^+/+^* and *hsd11b2^−/−^* 5-dpf larvae was further confirmed with the *Tg(9xGCRE-HSV.Ul23:EGFP)^ia20^* transgenic line, which allows the in vivo visualization of cortisol-dependent transcription through the production of EGFP [32]. As shown in Figure 5A and B, the intensity of fluorescence observed in *hsd11b2^+/+^;Tg(9xGCRE-HSV.Ul23:EGFP)^ia20^* larvae was not significantly different compared to the fluorescence observed in *hsd11b2^−/−^; Tg(9xGCRE-HSV.Ul23:EGFP)^ia20^* siblings.

### 2.3. Cortisol Stress Response in Adults

**Figure 6:**
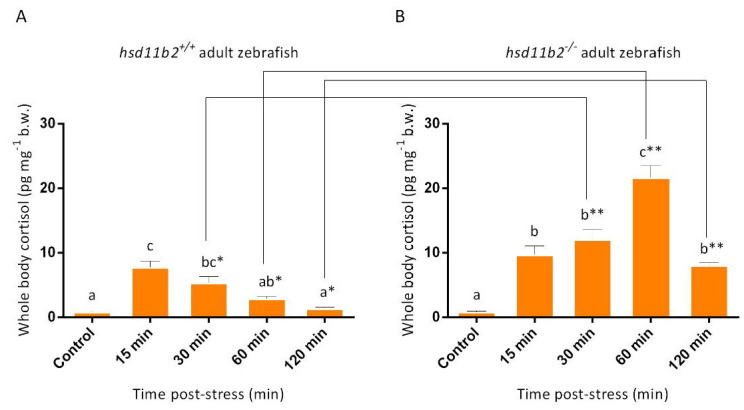
Effect of acute stress application on whole-body cortisol concentrations of one-year-old adult zebrafish, as depicted by bar graphs. Measurements of 10 *hsd11b2^+/+^* (**A**) WT and 10 *hsd11b2^−/−^* (**B**) mutant zebrafish prior to (control) at 15 min, 30 min, 60 min and 120 min after the stress application. Values are means ± SEM. Means with different letters differ significantly from one another (*p* < 0.001). Asterisks show statistically significant differences between the two groups (*p* < 0.001). Holm-Sidak comparison test was applied to identify groups that were significantly different. * *p* < 0.05, ** *p* < 0.01.

The cortisol stress response after an acute stress application was evaluated with one-year-old WT (*hsd11b2^+/+^*) and mutant (*hsd11b2^−/−^*) zebrafish as depicted in Figure 6. To this end, adult zebrafish of both groups were subjected to an acute stress and whole-body cortisol was measured prior to (0 min) stress exposure and at 15 min, 30 min, 60 min and 120 min after application of the stressor. For the *hsd11b2^+/+^* group, a statistically significant (*p* < 0.001) increase in whole-body cortisol concentrations was observed at 15 min post stress. More specifically, basal values of cortisol at time 0 (0.8 pg/mg ± 0.04 SEM) increased showing a peak at 15 min post stress (7.8 pg/mg ± 0.3 SEM), and then gradually decreased at 30 min (5.3 pg/mg ± 0.3 SEM) and 60 min (2.9 pg/mg ± 0.09) to return to basal levels at 120 min post stress (1.4 pg/mg ± 0.7 SEM) (Figure 6). On the other hand, for the *hsd11b2^−/−^* mutant group, a statistically significant (*p* < 0.001) increase was observed at 15 min post-stress (9.8 pg/mg ± 0.4 SEM). Then, cortisol concentrations continued to rise at 30 min post stress (12.1 pg/mg ± 0.5 SEM), showing peak values at 60 min post stress (21.7 pg/mg ± 0.6 SEM), with a tendency to return to basal levels observed at 120 min post stress (8.0 pg/mg ± 0.15) (Figure 6; *p* < 0.001).

### 2.4. Locomotor Response under Alternating Light/Dark Periods during Dark to Light Transition

**Figure 7:**
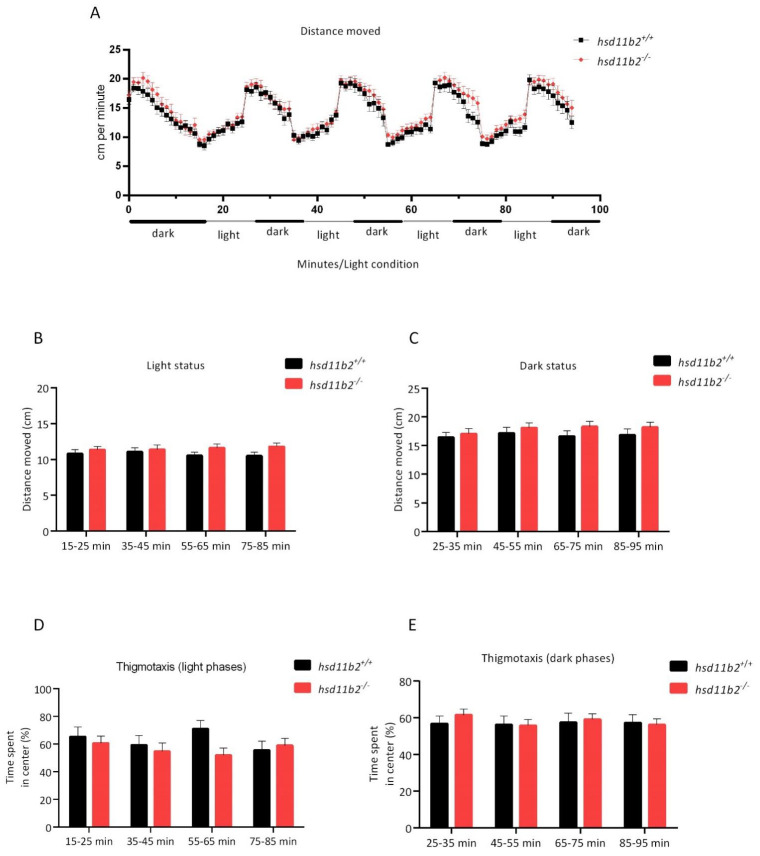
(**A**) Overview of the locomotor activity of zebrafish larvae of the two genotypes during four alternating phases of 0% and 5% light (139 lux). (**B**) Distance moved by zebrafish larvae of the two genotypes during four phases of 5% light (139 lux). (**C**) Distance moved by zebrafish larvae of the two genotypes during four phases of 0% light. (**D**,**E**) Percentage of the time spent in the “center” zone as an assessment of thigmotaxis (**D**) during four phases of 5% light and (**E**) four phases of 0% light. No statistically significant difference was observed. Holm-Sidak comparison test was applied to identify groups that were significantly different.

*hsd11b2^−/−^* larvae exhibited no statistically significant differences in levels of the locomotor activity compared to *hsd11b2^+/+^* individuals, both under light and under dark conditions. Figure 7 shows an overview of the locomotor activity of zebrafish larvae of the two genotypes during four alternating phases of light and dark (Figure 7A). For both genotypes, as the sequences of light and dark alterations advanced, the locomotor activity remained stable, showing higher locomotor activity in the dark (Figure 7B,C). The two experimental groups showed no differences in the time spent in the central zone, neither during the light or the darkness periods (Figure 7D,E).

### 2.5. Vibrational Startle Response Assay (VSRA)

**Figure 8:**
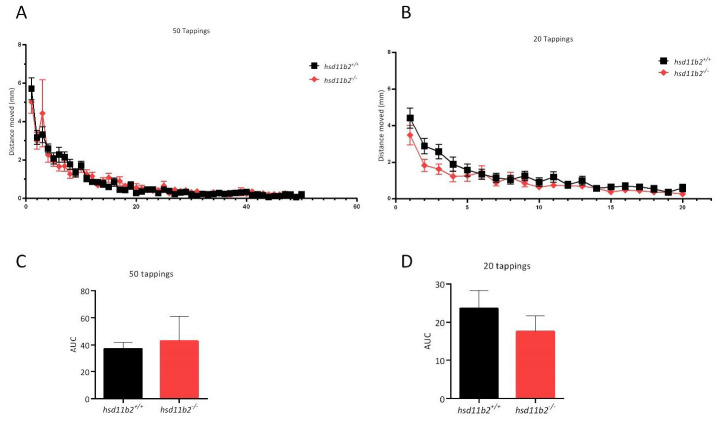
Effect of the VSRA on the escape response of zebrafish larvae. (**A**) Plots of mean distance moved ± SEM against 50 tapping stimuli at 1 s ISI. (**B**) Plots of mean distance moved ± SEM against a second sequence of 20 tapping stimuli at 1 s ISI (15 min after the 51st stimulus of the previous sequence). (**C**,**D**) Bar graphs of calculated AUC corresponding to the plots of graphs (**A**,**B**), respectively. Holm-Sidak comparison test was applied to identify groups that were significantly different.

The startle response of the larvae evoked by the first vibrational stimulus showed no statistically significant differences in the *hsd11b2^−/−^* larvae compared to *hsd11b2^+/+^* larvae as seen in Figure 8. The AUC calculations showed no statistically significant difference between the *hsd11b2^+/+^* and the *hsd11b2^−/−^* group either in the first sequence of 50 taps, nor in the second series of vibrational stimuli (20 taps) after 15 min of rest (Figure 8).

## 3. Discussion

It is well established that the coordinated HPI axis regulation of cortisol stress response commences only after hatch in zebrafish [9,10]. An important role in HPI regulation of the stress response is played by the Hsd11b2 enzyme, which converts active GCs into their inactive derivatives (e.g., cortisol to cortisone). Despite the growing number of studies on stress in zebrafish, little is known about how the deactivation of Hsd11b2 affects larval development, stress response, and behavior other than stress response, which was analyzed by Alderman and Vijayan by inhibition of Hsd11b2 with 18β-glycyrrhetinic acid, a well-known inhibitor of this enzyme [33].

In this work, we generated a zebrafish line bearing a homozygous inactivating mutation in the *hsd11b2* gene. This was achieved by CRISPR/Cas9 insertion of a 19-nucleotide segment, which resulted in a frameshift and lead to a premature stop codon. As determined by RT-qPCR analysis, the *hsd11b2* transcript levels were reduced by about 60% in mutants, likely due to the NMD machinery [31], a surveillance pathway that prevents truncated protein formation. The mutant line is viable but presents reduced life survival during larval stages and impairment of reproductive capabilities, with those of males being almost completely abolished. This is not surprising, given that this enzyme is involved in the synthesis of 11-ketotestosterone (11-KT), which is the main active form of androgens in zebrafish [17]. The loss of this enzyme can also lead to an imbalance of other steroid hormones, including testosterone (T) accumulation or loss of other intermediate molecules, which in turn can affect the expression levels of associated enzymes such as 11β-hydroxylase and 17β-hydroxysteroid dehydrogenase type 3 [34].

Androgen deficiency also results from the loss of the Cyp11c1 activity, together with the lack of cortisol production [35,36]. The occurrence of the testis in the two recently generated mutant lines demonstrates that androgens are not required for the development of the male gonad. However, 11-KT is required for sperm duct morphogenesis and promotes spermatogenesis and proper breeding behavior. In this study, we focused our attention on the possible effects of the knockout of this gene in whole-body cortisol concentration and stress response. The role of Hsd11b2 in reproduction requires a deeper analysis of all the steroid hormones and steroidogenic enzymes involved in this process and is in progress by our group.

Homozygous mutants measured at 6 dpf displayed a significant body length increase when compared to *hsd11b2^+/+^* siblings. These differences cannot be straightforwardly interpreted, as basal cortisol levels—which could positively account for differences in larval development [37]—are the same for the two genotypes at 5 dpf. However, as revealed by previous studies of whole-body cortisol levels during early developmental stages of teleosts, the maternal cortisol that is deposited into the oocyte prior to spawning and fertilization, tends to diminish as development proceeds and the de novo cortisol synthesis starts only after hatching [10,15,38,39]. The differences in growth observed at 6 dpf between the two genotypes may be accounted for by molecular events related to the different rate of depletion of maternal cortisol, due to the knockout of the *hsd11b2* gene in the mutant line. The Hsd11b2 enzyme is expressed since the first stages of development and its combined action with the 20β-Hydroxysteroid dehydrogenase type 2, that converts cortisone into 20β-hydroxycortisone, may be responsible for the reduction in cortisol maternal deposit during early development [40].

We then analyzed the effects of Hsd11b2 deactivation on zebrafish larvae, in terms of stress response and behavior. To this end, larvae at 5 dpf (both *hsd11b2^+/+^* and *hsd11b2^−/−^)* were exposed to an acute stress and the temporal trend of cortisol concentration was determined in individual larvae, based on the protocol developed by Samaras and Pavlidis [25]. The results of this experiment showed that *hsd11b2^+/+^* sibling larvae release cortisol with a peak 10 min post-stress and already 15 min after stress the cortisol returns to basal levels. This result with *hsd11b2^+/+^*, agree with previous studies that have examined the temporal pattern of cortisol release after an acute stressor even though it has differences in onset of response, differences that could be due to changes in the duration of the applied stress [11,12,13].

Conversely, although *hsd11b2^−/−^* larvae show no statistically significant differences in the basal cortisol levels when compared to *hsd11b2^+/+^* siblings, they show a higher magnitude (peak at 10-min post stress) and a more prolonged acute stress response over time with a cortisol peak lasting up to 30 min after the stress event with respect to their *hsd11b2^+/+^* siblings. Additionally, similar results were obtained with adults, as *hsd11b2^−/−^* adult zebrafish showed a prolonged cortisol response characterized also by higher cortisol levels when compared to the *hsd11b2^+/+^* siblings. These results are in agreement with those obtained by Alderman and Vijayan [33], who described, after an acute stress, an increase and longer lasting whole-body cortisol levels after inhibition of Hsd11b2 activity by 18β-glycyrrhetinic acid, an inhibitor of this enzyme [41], suggesting an important role of the Hsd11b2 enzyme in the recovery of physiological condition after stress.

Although cortisol catabolism was delayed, the mutant larvae and adults eventually managed to reduce their stressed-induced high levels of cortisol. Two hours after stress, mutant larvae reached basal cortisol levels whereas, in adult mutants, this seems to require more time. However, the mechanism that allows cortisol catabolism in absence of Hsd11b2 activity needs to be analyzed, focusing the attention on other pathways that are known to lead to cortisol inactivation. As reported by Shiffer and collaborators [42] and Tokarz and collaborators [34], apart from Hsd11b2, which represents the principal enzyme involved in cortisol catabolism, deactivation of this hormone can be obtained also by the activity of 20β-hydroxysteroid dehydrogenase (Hsd20b2), 5α-reductase type 1 and 2 (Srd5a1/2), 3β-hydroxysteroid dehydrogenase (Hsd3b1), or by further downstream enzymes such as UDP-glucuronosyltransferases and sulfotransferases. Thus, expression of these enzymes, and especially a possible up-regulation, would be of high interest for future studies.

The understanding that zebrafish larvae are sensitive to various stimuli, including vision, touch, audition, chemosensation, vestibular inputs and heat [43], stimulated the design and implementation of several behavioral protocols. Larval behavioral responses have been well characterized [44,45], while the number of behaviors studied in zebrafish larvae has been increasing throughout the years, from locomotor and escape/startle responses, to phototaxis and hunting/feeding [46]. One of the protocols developed for the analysis of the behavioral phenotype of zebrafish mutant lines is that of the Locomotor Response under alternating Light/Dark periods (LMR-L/D). Locomotor activity of zebrafish during periods of bright illumination is thought to be decreased, whereas the onset of darkness induces an increase in motor activity [25,26,27].

In our study, the results of the LMR-L/D behavioral experiment are in accordance with those referred in literature, where zebrafish larvae exhibit a specific pattern of movement when exposed to a protocol of light-dark alteration [47,48]. More specifically, the transition from dark to light decreases locomotor activity, while the transition from light to dark increases locomotion, with this specific rise being commonly attributed to the increased stress/anxiety level in zebrafish larvae. In our study, both *hsd11b2^+/+^* and *hsd11b2^−/−^* larvae exhibited similar locomotor activity when exposed to alternating light/dark conditions. Larvae of both genotypes exposed to light exhibited a decreased locomotor activity when compared to dark environment, with no statistically significant differences observed for the two groups. During the VSRA assay, we examined the escape response and habituation in zebrafish larvae, but the initial startle response in both sequences of stimuli showed no statistically significant differences in neither of the two series of tappings between the two genotypes.

The results regarding the similar behavior of the two genotypes during the two behavioral tests may be interpreted by the fact that both genetic lines exhibit similar basal levels of cortisol and therefore the factor that could induce a behavioral difference is absent in the non-stressed state of the larvae. All in all, the behavioral experiments faced the limitation of a possible missed effect of the differentiated cortisol response between the two groups, as the prolonged cortisol response in the *hsd11b2^−/−^* group may have effects on behavior. Future work would include behavioral experiments for larvae and adults (both WT and the mutant line) carried out after an acute stress challenge and within the time period that the prolonged peak in cortisol is observed in *hsd11b2^−/−^* mutants.

This study investigated *hsd11b2* silencing in both zebrafish larvae and adults for the first time, revealing a prolonged cortisol stress response characterized by significantly higher levels of cortisol and a delay in returning to baseline levels which support the evidence that Hsd11b2 plays, in zebrafish, a critical role in the negative feedback regulation of cortisol during stress. The *hsd11b2* mutation had no effect on the behavior of zebrafish larvae-based of the locomotor activity during alternating light/dark periods and after a vibrational stimulus, probably reflecting a minor role of this enzyme in the regulation of the basal cortisol levels of unstressed fish. A proposed future study would include a revision of the larval behavioral experiments using stressed larvae, in order to reveal whether the observed prolonged cortisol response of the mutant line has an effect on their behavior.

Apart from the role on stress response, this mutant line would help to shed light on the role of the *hsd11b2* gene in other physiological processes, especially regarding the reproductive process and the regulation of cortisol catabolism.

## 4. Materials and Methods

### 4.1. Adult Zebrafish Husbandry and Breeding

WT zebrafish (*Danio rerio*) of the Tubingen line were maintained according to standard procedures in ZebTec Active Blue–Stand Alone systems (Tecniplast, Varese, Italy) at the Animal House facilities of the Fish Physiology Laboratory, University of Crete and at the Zebrafish facility of University of Padova and used to generate the mutant line. In both facilities, zebrafish husbandry systems were equipped with biological and a self-cleaning drum filter. The system had a constant temperature of 28.0 °C under a photoperiod of 12L:12D. Fish were fed twice a day with commercial feed (Zebrafeed, Sparos I&D, Portugal) and live Artemia. The mutant line was also prepared on *Tg(9xGCRE-HSV.Ul23:EGFP)^ia20^* transgenic background, for in vivo visualization of cortisol-dependent transcription through the production of EGFP [32].

Breeding was performed in special spawning devices; embryos were collected and placed in 500 mL water tanks (200 eggs L^−1^). Embryos and larvae were reared until 5 dpf and used either for the acute stress or the behavioral experiments.

### 4.2. Generation of hsd11b2 Zebrafish Mutant Line

The generation of the *hsd11b2* mutant zebrafish line was performed using the CRISPR/Cas9-mediated genome editing, at the University of Padova. Briefly, the CHOPCHOP (available at https://chopchop.rc.fas.harvard.edu) and the Breaking-Cas (available at https://bioinfogp.cnb.csic.es/tools/breakingcas/) software packages were used to design the gene-specific guide RNA (sgRNA) (CGGTGCCATGCCCTCAGTGGTGG) to target *hsd11b2* gene on exon 1 (AC NM131479). The guide was subsequently purchased from Synthego (Menlo Park, CA, USA). Fertilized eggs were injected with 1 nL of a solution containing 280 ng/μL of Cas9 (M0386T, New England Biolabs, Ipswich, MA, USA)) and 3 pmol/μL of *hsd11b2*-targeting sgRNA. Genomic DNA was extracted from 5-dpf injected larvae to test the presence of mutations and to confirm the activity of the Cas9 enzyme. Injected embryos were raised to adulthood and screened, by F1 genotyping, for germline transmission of the mutation. A F1 mutant carrier harboring an insertion of 19 nucleotides was selected and bred with WT to obtain the F2 generation. The resulting heterozygous F2 individuals were incrossed, to obtain F3 generation that includes homozygous mutants.

### 4.3. Genomic DNA Extraction and Genotyping

Genomic DNA was extracted from single larvae euthanized with tricaine overdose (0.3 mg/mL) (MS222; Sigma-Aldrich, E10521, Milan, Italy) using the HotSHOT protocol [49]. For adult genotyping, the fish were anesthetized with tricaine (0.16 mg/mL) and a small fragment of tissue was removed from the caudal fin with a sharp blade. The specimens were then treated with the same HotSHOT protocol.

Mutations in F1 were detected by means of heteroduplex mobility assay (HMA). Briefly, genomic fragments corresponding to the target site were amplified by PCR with 5x HOT FIREPol^®^ Blend Master Mix (Solis BioDyne, 04-25-00125, Tartu, Estonia) and the locus-specific primers (*hsd11b2*-F1 and *hsd11b2*-R1, listed in Table 1), that gave rise to a 187-bp fragment on WT samples. PCR conditions were as follows: 15 min at 95 °C, 35 cycles at 95 °C for 20 s, 60 °C for 30 s and 72 °C for 30 s. The resulting PCR amplicons were subjected to electrophoresis on a 15% polyacrylamide gel (ThermoFisher Scientific, NP0323PK2, Waltham, MA, USA). To identify optimal mutation, PCR products from fish harboring indel mutations were sequenced. Poly Peak Parser software (http://yosttools.Genetics.utah.edu/PolyPeakParser/) was used for identification and sequence characterization of heterozygous mutant carriers generated by genome editing. The same primers used for HMA on F1 and F2 samples were used to screen homozygous and heterozygous animals. PCR products were resolved with GelRed-stained 2% agarose low EEO gel (Fisher BioReagents, Waltham, MA, USA, BP160-500) to identify *hsd11b2^+/+^*, *hsd11b2^+/−^* and *hsd11b2^−/−^* samples. Primer sequences are listed in Table 1.

### 4.4. mRNA Isolation and RT-qPCR

RNA was obtained from pools of at least 15 larvae at 5 dpf with TRIZOL reagent (Thermo Fisher Scientific, 15596018, Waltham, MA, USA). After treatment with RQ1 RNase-free DNase (Promega, M6101, Madison, WI, USA) to eliminate DNA genomic contamination, total RNA was retrotranscribed with FIREscript RT cDNA synthesis mix (Solis Biodyne, 06-13-0000S) according to the manufacturer’s protocol. RT-qPCRs were performed in triplicate with the CybrGreen method using CFX384 Touch Real-Time PCR Detection System (Bio-Rad, Hercules, CA, USA) and the 5xHOT FIREpol EvaGreen qPCR mix plus (Solis Biodyne, 08-36-00001). Zebrafish *ef1α* was used as an internal standard in each sample to normalize the results and compensate variations in mRNA and cDNA quantity and quality.

Amplification program was composed of 15 min at 95 °C, followed by 45 cycles of 95 °C for 15 s, 60 °C for 20 s and 72 °C for 20 s. No amplification products were observed in negative controls and no primer–dimer formations in control templates. All analyses were performed in triplicate. Changes in gene expression were calculated with respect to the control, sampled at the same time of treatment. The primer sequences are reported in Table 1.

### 4.5. Morphometric Analyses

Larvae obtained from breeding between *hsd11b2^+/−^* adult fish were anesthetized with tricaine, embedded in 2% methylcellulose and mounted in a depression slide. Pictures were taken in bright field using Leica M165 FC microscope with a digital camera Leica DFC7000T. Pictures were then analyzed with ImageJ software. We measured full length from the head to the end of the trunk (caudal fin excluded), eye diameter (measuring two diagonals and then calculating the average of the diagonals) and eye area as reported in Tarasco et al. [50]. *hsd11b2;Tg(9xGCRE-HSV.Ul23:EGFP)^ia20^* larvae were mounted in a depression slide in 2% methylcellulose and pictures were taken with Leica M165 FC microscope equipped with a Nikon DS-Fi2 digital camera. Fluorescence analyses were performed with ImageJ software.

### 4.6. Behavioral Analysis

Larvae behavioral tests were performed in 24-well plates, during the same time frame each day (between 11:00 am and 4:00 pm), using larvae from heterozygous incrosses. In the end of every behavioral test, the larvae were euthanized with a tricaine overdose and genotyped. The behavior of the confirmed homozygous *hsd11b2^−/−^* mutant larvae was compared with that of *hsd11b2^+/+^* larvae.

#### 4.6.1. Locomotor Response under Alternating Light/Dark Periods (LMR-L/D)

This protocol aimed to study the behavioral effects in response to alternating dark and light conditions. For this purpose, 5-dpf larvae were individually transferred to clear 24-well plates with 1.5 mL of egg water medium. The following day, starting at 11:00 (after an overnight acclimatization) each plate was exposed to the light-dark protocol in the DanioVision Observation Chamber (DVOC-0040) (Noldus, Wageningen, The Netherlands). The protocol consisted of an initial 15 min 0% light condition (acclimatization to the environment in the chamber) and four alternating 10 min periods of 5% light and 0% light (dark conditions). Protocol’s total duration was 95 min. The chamber temperature was set to 28 °C. The movement of each larva was video captured and quantified using the Ethovision Software XT14 (Noldus). A round-shaped zone in the center of the arena (1.3 cm diameter) was defined as “center” for each well. The swimming activity was determined as the total swimming distance (mm) per larva and as the swimming distance (mm) per larva during light and dark conditions. In an effort to analyze the thigmotactic behavior, the time spent by each larva in the central zone was calculated according to the formula depicted below [48,51]: *Time spent in “center” zone = [time spent in “center” zone/duration of time bin] × 100.*

#### 4.6.2. Vibrational Startle Response Assay (VSRA)

The design of this protocol was based on already published studies [28,45]. The behavioral experiments were performed in the DanioVision Observation Chamber (DVOC-0040), coupled with the DanioVision Tapping Device DVTD-0010 that ensured the automatic delivery of the vibrational stimuli. The protocol consisted of sequences of vibrational stimuli during fixed time periods referred to as interstimulus interval (ISI). Vibrational stimulus was selected at the highest tapping intensity (intensity level: 8), while the chamber temperature was set to 28 °C. The 5-dpf larvae were transferred individually into 24-well plates containing 1.5 mL of egg water medium and were left undisturbed for 24 h. The next day, each 24-well plate was transferred into the DanioVision Observation Chamber, and after an acclimation period of 15 min, 50 tapping stimuli were delivered at 1 s ISI. The recovery of the tapping startle was elicited by applying a second session of 20 tapping stimuli (1 s ISI) 15 min after the 50th tap of the first session. Videos were recorded at 25 frames per second and the VSRA was analyzed for each individual larva by measuring the distance moved (mm) over the 1 s period after every tapping stimulus. Startle response was measured as the total distance moved (mm) in response to the first tapping stimulus, whereas habituation was assessed by analyzing the area under the curve (AUC) of the plots of distance moved (mm) for the two sequences of stimuli.

### 4.7. Acute Stress Protocol in Larvae

The 5-dpf larvae were subjected to an acute stress which consists of osmotic stress at 250 mM NaCl for 3 min, as described by Yeh and coworkers [12], followed by air exposure for 20 s. After the short air exposure larvae were transferred to clean water and samples were collected at the following time points: 5 min, 10 min, 15 min, 30 min, 60 min and 120 min after stress application. Samples were also collected before applying the stress and served as controls (0 min). The collected samples (*n* = 60 for each time point) were euthanized instantly in tricaine overdose and transferred individually in tubes to −20 °C upon genotyping and cortisol analysis.

### 4.8. Acute Stress Protocol in Adults

Adult fish (1 year old) of the two different genotypic groups (*hsd11b2^+/+^*, *hsd11b2^−/−^*) were subjected to an acute stress which consisted of chasing for 3 min and air exposure for 1 min. After the stress application fish were transferred to clean water tanks and 10 samples were collected at the following time points: 15 min, 30 min, 60 min and 120 min after stress application. 10 samples were collected prior to the application of the stress and served as controls. The collected samples were euthanized instantly in tricaine overdose (MS-222, 0.3 g/L) and transferred individually in tubes to −20 °C upon cortisol analysis.

### 4.9. Cortisol Extraction from Individual Larvae

Cortisol extraction from larva samples was performed according to Samaras and Pavlidis [25]. Individual larvae samples were placed in plastic round-bottom tubes with 100 μL phosphate buffer and were homogenized with a bead mill disruptor (Tissue Lyser II, Qiagen) for 2 min at 30 Hz using small sized beads (3 mm) at room temperature. Immediately after the homogenization, 1 μL of the homogenate was removed for genotypic analysis and the rest 99 μL were transferred to a glass tube adding 1 mL of diethyl-ether and vortexed. The glass tube was subsequently placed in −80 °C and the separated diethyl ether layer was transferred into a new glass tube. Diethyl ether was let to evaporate completely in a 45 °C water bath for 20 min. Samples (*n* = 10 from each time point and each genotypic group) were reconstituted in 100 μL of the enzyme immunoassay extraction buffer. Cortisol concentration was quantified with a commercial enzyme immunoassay kit (Cortisol ELISA kit, Neogen Corporation, Lansing, MI, USA). In this kit, the antibody used shows different degrees of cross-reactivity with other closely related steroids like cortisone (15.7%), 11-deoxycortisol (15%), corticosterone (4.8%) and prednisone (7.8).

### 4.10. Whole-Body Cortisol Extraction from Adults

Individual whole-body samples were weighed and homogenized with 5 (*w*/*v*) volume per body weight of phosphate buffered saline (pH 7.4) using a rotor homogenizer, according to de Jesus et al. [52] and Pavlidis et al. [53]. A total volume of 500 μL of the homogenate was vortexed in a glass tube with 2 mL of diethyl-ether. The diethyl ether layer was allowed to separate in −80 °C and then transferred to a new glass tube, which was placed in a 45 °C water bath for 20 min to allow the diethyl ether to evaporate. Samples were reconstituted in 100 μL of the enzyme immunoassay extraction buffer. Cortisol concentration was quantified with a commercial enzyme immunoassay kit (Cortisol, ELISA kit, Neogen Corporation, Lansing, MI, USA). The cross-reactivity of the antibody with other related steroids is the same as mentioned above.

### 4.11. Histological Analysis

*hsd11b2^+/−^* and *hsd11b2^−/−^* adult fish (8 mpf) were fixed in Bouin’s solution for 24 h at room temperature. The samples were dehydrated through a graded series of ethanol solutions and embedded in Paraplast plus (Leica, 39602004, Wetzlar, Germany). The samples were serially cut into 7/8-µm sections on a LKB microtome. After rehydration, the sections were stained with hematoxylin and eosin and mounted with Eukitt (BioOptica, 09-00100, Milano, Italy) for microscopic examination.

### 4.12. Statistical Analysis

Data were analyzed with IBM SPSS 22 (IBM, Chicago, IL, USA), while GraphPad Prism 8 (GraphPad Software, USA) was used to generate the graphs presented. All data are presented as mean ± standard error of the mean (SEM), unless otherwise stated. Significance was set at *p* < 0.05. For the *hsd11b2^+/+^* group and for each knockout mutant line group, statistical comparisons of temporal patterns of cortisol prior to and at the different time points after the application of the acute stress were made using one way ANOVA. A two-way ANOVA was used to analyze the data from the different light periods of the two genetic lines, whereas total combined distance moved (mm) in the light and in the dark periods of the LMR-L/D test was analyzed by Student’s *t*-test. Regarding the data obtained from the VSRA, distance travelled by the WT and *hsd11b2^−/−^* larvae for each stimulus delivered was compared using Student’s *t*-test. The area under the curve (AUC) was calculated using Y = 0 and peaks less than 10% of the distance from minimum to maximum Y were ignored. If significant (*p* < 0.05), Holm-Sidak comparison test was applied to identify groups that were significantly different.

## Figures and Tables

**Table 1:** List of primers used in the corresponding application.

Gene Name	Sequence (5′→3′)	AC Number	Application
*hsd11b2*-F1	GTCTTTCTGGATTTACATCGGC	NM_212720	genotyping
*hsd11b2*-R1	GCAGCACACACAGAG	NM_212720	genotyping
*hsd11b2*-F	GTCCTCTGTGTGTGCTGC	NM_212720	qPCR
*hsd11b2*-R	GCTTGCTGTACCTGCTGAG	NM_212720	qPCR
*ef1α*-F	TTCGAGAAGGAAGCCGCTG	AY422992	qPCR
*ef1α*-R	CAGCAACAATCAGCACAGCAC	AY422992	qPCR

## Data Availability

Not applicable.
